# 4,4′-Dimethyl-1,1′-[ethyl­enedioxy­bis(nitrilo­methyl­idyne)]dibenzene

**DOI:** 10.1107/S1600536809015840

**Published:** 2009-05-07

**Authors:** Yu-Jie Ding, Zhu-Lian Xue, Wen-Kui Dong, Yin-Xia Sun, Jian-Chao Wu

**Affiliations:** a Department of Biochemical Engineering, Anhui University of Technology and Science, Wuhu 241000, People’s Republic of China; b School of Chemical and Biological Engineering, Lanzhou Jiaotong University, Lanzhou 730070, People’s Republic of China

## Abstract

The Schiff base, C_18_H_20_N_2_O_2_, which lies about an inversion centre, adopts a linear conformation. The mol­ecules are packed by C—H⋯π inter­actions, forming a two-dimensional supra­molecular network.

## Related literature

For background literature on Schiff base oximes, see: Akine *et al.* (2005 ▶); Dong *et al.* (2008, 2009*a* ▶,*b* ▶); Yamada (1999 ▶). For a related structure, see: Dong *et al.* (2008 ▶).
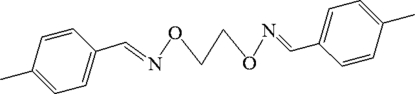


## Experimental

### 

#### Crystal data

C_18_H_20_N_2_O_2_
*M* *_r_* = 296.36
Monoclinic, 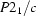

*a* = 13.6946 (12) Å
*b* = 4.8196 (9) Å
*c* = 12.1644 (11) Å
β = 104.936 (1)°
*V* = 775.75 (17) Å^3^
*Z* = 2
Mo *K*α radiation
μ = 0.08 mm^−1^
*T* = 298 K
0.43 × 0.20 × 0.10 mm

#### Data collection

Siemens SMART 1000 CCD area-detector diffractometer
Absorption correction: none
3790 measured reflections
1370 independent reflections
1012 reflections with *I* > 2σ(*I*)
*R*_int_ = 0.043

#### Refinement

*R*[*F* ^2^ > 2σ(*F* ^2^)] = 0.051
*wR*(*F* ^2^) = 0.158
*S* = 1.03
1370 reflections
100 parameters
H-atom parameters constrained
Δρ_max_ = 0.23 e Å^−3^
Δρ_min_ = −0.21 e Å^−3^

### 

Data collection: *SMART* (Siemens, 1996 ▶); cell refinement: *SAINT* (Siemens, 1996 ▶); data reduction: *SAINT*; program(s) used to solve structure: *SHELXS97* (Sheldrick, 2008 ▶); program(s) used to refine structure: *SHELXL97* (Sheldrick, 2008 ▶); molecular graphics: *SHELXTL* (Sheldrick, 2008 ▶); software used to prepare material for publication: *SHELXTL*.

## Supplementary Material

Crystal structure: contains datablocks global, I. DOI: 10.1107/S1600536809015840/ng2575sup1.cif

Structure factors: contains datablocks I. DOI: 10.1107/S1600536809015840/ng2575Isup2.hkl

Additional supplementary materials:  crystallographic information; 3D view; checkCIF report

## Figures and Tables

**Table 1:** Hydrogen-bond geometry (Å, °)

*D*—H⋯*A*	*D*—H	H⋯*A*	*D*⋯*A*	*D*—H⋯*A*
C9—H9*A*⋯*Cg*1	0.96	2.66	3.578 (2)	160

*Cg*1 is the centroid of the C3–C8 ring.

**Fig. 1.**
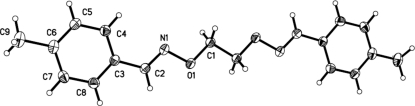
The molecular structure of the title compound with atom numbering scheme [Symmetry codes: -x + 2,-y,-z + 1]. Displacement ellipsoids for non-hydrogen atoms are drawn at the 30% probability level.

**Fig. 2.**
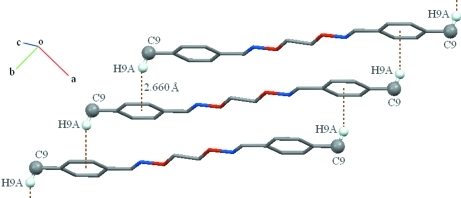
Part of the supramolecular structure of the title compound. C—H···π(Ph) interactions are shown as dashed lines.

## supplementary crystallographic information

### Comment

Schiff bases and their bis-oxime analogues are a significant class of compounds which can be used in a variety of studies such as organic synthesis, catalyst, drug design and life science and so on (Yamada, 1999; Akine *et al.*, 2005; Dong *et al.*, 2009*a*). In order to extend our work (Dong *et al.*, 2008) on structural characterization of bisoxime compounds, we report the synthesis and the X-ray structure of 4,4'-dimethyl-1,1'-[ethylenedioxybis(nitrilomethylidyne)]dibenzene in this paper (Fig. 1).

The molecule of the title compound is disposed about a crystallographic inversion centre (Symmetry codes: -*x*, -*y*,-*z*) and twofold screw axis (symmetry code: -*x*, 1/2 + *y*, 1/2 - *z*). The oxime, methyl groups and benzene rings have anti-conformation. The two benzene rings of the molecule are parallel, and the methyl and oxime (–CH_2_—O—N=C–) functional groups are coplanar with the benzene ring in each half of the molecule.

The molecule adopts a linear-shaped configuration with respect to the oxime C=N bonds, which is different from our previous reported bisoxime of 3,3'-dibromo-1,1'-[ethylenedioxybis(nitrilomethylidyne)]dibenzene in which the molecule assumes an E configuration (Dong *et al.*, 2008). The packing of the molecule is controlled by C—H···π(Ph) interactions linking molecules into infinite supramolecular structure along *b* axis (Fig. 2).

### Experimental

4,4'-Dimethyl-1,1'-[ethylenedioxybis(nitrilomethylidyne)]dibenzene was synthesized according to an analogous method reported earlier (Dong *et al.*, 2009*b*). To an ethanol solution (4 ml) of 4-methyl-2-hydroxybenzaldehyde (125.8 mg, 1.05 mmol) was added an ethanol solution (3 ml) of 1,2-bis(aminooxy)ethane (47.7 mg, 0.518 mmol). The reaction mixture was stirred at 328–333 K for 8 h. After cool to room temperature, no precipitate was formed, which was concentrated to about 1 ml under reduced pressure. The precipitate formed was separated by filtration, and washed several times with n-hexane. The product was dried under vacuum to yield 90.0 mg of the title compound. Yield, 58.6%. mp. 359–360 K. Anal. Calcd. for C_18_H_20_N_2_O_2_: C, 72.95; H, 6.80; N, 9.45. Found: C, 72.66; H, 6.87; N, 9.32.

Colorless needle-like single crystals suitable for X-ray diffraction studies were obtained after about four days by slow evaporation from an diethyl ether solution of the title compound.

### Refinement

Non-H atoms were refined anisotropically. H atoms were treated as riding atoms with distances C—H = 0.97 (CH_2_), 0.93 Å (CH), C—H = 0.96 (CH_3_) Å and *U*_iso_(H) = 1.2 *U*_eq_(C) and 1.5 *U*_eq_(O).

### Crystal data

**Table:**

C_18_H_20_N_2_O_2_	*F*(000) = 316
*M**_r_* = 296.36	*D*_x_ = 1.269 Mg m^−^^3^
Monoclinic, *P*2_1_/*c*	Mo *K*α radiation, λ = 0.71073 Å
Hall symbol: -P 2ybc	Cell parameters from 1400 reflections
*a* = 13.6946 (12) Å	θ = 3.4–27.7°
*b* = 4.8196 (9) Å	µ = 0.08 mm^−^^1^
*c* = 12.1644 (11) Å	*T* = 298 K
β = 104.936 (1)°	Column, colorless
*V* = 775.75 (17) Å^3^	0.43 × 0.20 × 0.10 mm
*Z* = 2	

### Data collection

**Table:**

Siemens SMART 1000 CCD area-detector diffractometer	1012 reflections with *I* > 2σ(*I*)
Radiation source: fine-focus sealed tube	*R*_int_ = 0.043
graphite	θ_max_ = 25.0°, θ_min_ = 1.5°
φ and ω scans	*h* = −16→15
3790 measured reflections	*k* = −5→5
1370 independent reflections	*l* = −14→13

### Refinement

**Table:**

Refinement on *F*^2^	Primary atom site location: structure-invariant direct methods
Least-squares matrix: full	Secondary atom site location: difference Fourier map
*R*[*F*^2^ > 2σ(*F*^2^)] = 0.051	Hydrogen site location: inferred from neighbouring sites
*wR*(*F*^2^) = 0.158	H-atom parameters constrained
*S* = 1.03	*w* = 1/[σ^2^(*F*_o_^2^) + (0.1037*P*)^2^] where *P* = (*F*_o_^2^ + 2*F*_c_^2^)/3
1370 reflections	(Δ/σ)_max_ = 0.001
100 parameters	Δρ_max_ = 0.23 e Å^−^^3^
0 restraints	Δρ_min_ = −0.21 e Å^−^^3^

### Special details

**Table:**

Geometry. All e.s.d.'s (except the e.s.d. in the dihedral angle between two l.s. planes) are estimated using the full covariance matrix. The cell e.s.d.'s are taken into account individually in the estimation of e.s.d.'s in distances, angles and torsion angles; correlations between e.s.d.'s in cell parameters are only used when they are defined by crystal symmetry. An approximate (isotropic) treatment of cell e.s.d.'s is used for estimating e.s.d.'s involving l.s. planes.
Refinement. Refinement of *F*^2^ against ALL reflections. The weighted *R*-factor *wR* and goodness of fit *S* are based on *F*^2^, conventional *R*-factors *R* are based on *F*, with *F* set to zero for negative *F*^2^. The threshold expression of *F*^2^ > σ(*F*^2^) is used only for calculating *R*-factors(gt) *etc*. and is not relevant to the choice of reflections for refinement. *R*-factors based on *F*^2^ are statistically about twice as large as those based on *F*, and *R*- factors based on ALL data will be even larger.

### Fractional atomic coordinates and isotropic or equivalent isotropic displacement parameters (Å^2^)

**Table:**

	*x*	*y*	*z*	*U*_iso_*/*U*_eq_	
N1	0.87170 (11)	0.4357 (3)	0.55939 (12)	0.0389 (4)	
O1	0.95570 (9)	0.2523 (3)	0.58336 (10)	0.0424 (4)	
C1	0.95420 (13)	0.0920 (4)	0.48465 (15)	0.0381 (5)	
H1A	0.9568	0.2120	0.4214	0.046*	
H1B	0.8931	−0.0187	0.4631	0.046*	
C2	0.86877 (13)	0.5688 (4)	0.64775 (16)	0.0386 (5)	
H2	0.9180	0.5342	0.7148	0.046*	
C3	0.79062 (13)	0.7762 (4)	0.64868 (15)	0.0366 (5)	
C4	0.71621 (13)	0.8512 (4)	0.55207 (15)	0.0417 (5)	
H4	0.7148	0.7693	0.4825	0.050*	
C5	0.64425 (14)	1.0474 (4)	0.55907 (16)	0.0439 (5)	
H5	0.5956	1.0971	0.4934	0.053*	
C6	0.64272 (13)	1.1717 (4)	0.66137 (17)	0.0434 (5)	
C7	0.71769 (15)	1.0973 (4)	0.75725 (16)	0.0457 (5)	
H7	0.7189	1.1784	0.8270	0.055*	
C8	0.79055 (14)	0.9047 (4)	0.75058 (15)	0.0424 (5)	
H8	0.8406	0.8602	0.8158	0.051*	
C9	0.56330 (16)	1.3823 (4)	0.6689 (2)	0.0577 (6)	
H9A	0.5911	1.5656	0.6709	0.086*	
H9B	0.5417	1.3507	0.7369	0.086*	
H9C	0.5065	1.3647	0.6038	0.086*	

### Atomic displacement parameters (Å^2^)

**Table:**

	*U*^11^	*U*^22^	*U*^33^	*U*^12^	*U*^13^	*U*^23^
N1	0.0315 (8)	0.0384 (9)	0.0465 (9)	0.0052 (7)	0.0093 (6)	0.0011 (7)
O1	0.0343 (8)	0.0455 (8)	0.0446 (8)	0.0100 (6)	0.0052 (6)	−0.0053 (6)
C1	0.0335 (10)	0.0397 (10)	0.0405 (10)	0.0010 (8)	0.0084 (8)	−0.0035 (8)
C2	0.0349 (10)	0.0398 (11)	0.0396 (10)	0.0016 (8)	0.0072 (8)	0.0006 (8)
C3	0.0324 (10)	0.0365 (10)	0.0414 (10)	−0.0013 (7)	0.0104 (8)	0.0016 (8)
C4	0.0393 (10)	0.0446 (11)	0.0415 (10)	−0.0007 (9)	0.0108 (8)	−0.0032 (8)
C5	0.0366 (10)	0.0462 (11)	0.0473 (11)	0.0040 (8)	0.0080 (8)	0.0058 (9)
C6	0.0398 (11)	0.0342 (10)	0.0604 (12)	0.0000 (9)	0.0204 (9)	0.0036 (9)
C7	0.0537 (12)	0.0417 (11)	0.0452 (11)	0.0029 (9)	0.0189 (9)	−0.0056 (9)
C8	0.0452 (11)	0.0419 (11)	0.0388 (10)	0.0018 (9)	0.0085 (8)	0.0006 (8)
C9	0.0510 (12)	0.0461 (12)	0.0816 (17)	0.0075 (10)	0.0276 (11)	0.0031 (12)

### Geometric parameters (Å, °)

**Table:**

N1—C2	1.261 (2)	C4—H4	0.9300
N1—O1	1.4203 (18)	C5—C6	1.386 (3)
O1—C1	1.424 (2)	C5—H5	0.9300
C1—C1^i^	1.503 (3)	C6—C7	1.388 (3)
C1—H1A	0.9700	C6—C9	1.508 (3)
C1—H1B	0.9700	C7—C8	1.380 (3)
C2—C3	1.466 (2)	C7—H7	0.9300
C2—H2	0.9300	C8—H8	0.9300
C3—C8	1.386 (2)	C9—H9A	0.9600
C3—C4	1.390 (3)	C9—H9B	0.9600
C4—C5	1.384 (2)	C9—H9C	0.9600
			
C2—N1—O1	110.10 (14)	C4—C5—H5	119.1
N1—O1—C1	109.26 (12)	C6—C5—H5	119.1
O1—C1—C1^i^	106.32 (17)	C5—C6—C7	117.62 (17)
O1—C1—H1A	110.5	C5—C6—C9	121.59 (19)
C1^i^—C1—H1A	110.5	C7—C6—C9	120.78 (19)
O1—C1—H1B	110.5	C8—C7—C6	120.92 (18)
C1^i^—C1—H1B	110.5	C8—C7—H7	119.5
H1A—C1—H1B	108.7	C6—C7—H7	119.5
N1—C2—C3	122.47 (17)	C7—C8—C3	121.35 (17)
N1—C2—H2	118.8	C7—C8—H8	119.3
C3—C2—H2	118.8	C3—C8—H8	119.3
C8—C3—C4	118.06 (17)	C6—C9—H9A	109.5
C8—C3—C2	118.70 (17)	C6—C9—H9B	109.5
C4—C3—C2	123.24 (17)	H9A—C9—H9B	109.5
C5—C4—C3	120.27 (17)	C6—C9—H9C	109.5
C5—C4—H4	119.9	H9A—C9—H9C	109.5
C3—C4—H4	119.9	H9B—C9—H9C	109.5
C4—C5—C6	121.76 (17)		
			
C2—N1—O1—C1	176.47 (14)	C4—C5—C6—C7	1.4 (3)
N1—O1—C1—C1^i^	178.87 (16)	C4—C5—C6—C9	−179.11 (16)
O1—N1—C2—C3	179.41 (14)	C5—C6—C7—C8	−0.5 (3)
N1—C2—C3—C8	177.36 (16)	C9—C6—C7—C8	−179.96 (17)
N1—C2—C3—C4	−2.7 (3)	C6—C7—C8—C3	−0.9 (3)
C8—C3—C4—C5	−0.5 (3)	C4—C3—C8—C7	1.4 (3)
C2—C3—C4—C5	179.58 (16)	C2—C3—C8—C7	−178.65 (16)
C3—C4—C5—C6	−0.9 (3)		

Symmetry codes: (i) −*x*+2, −*y*, −*z*+1.

### Hydrogen-bond geometry (Å, °)

**Table:**

*D*—H···*A*	*D*—H	H···*A*	*D*···*A*	*D*—H···*A*
C9—H9A···Cg1	0.96	2.66	3.578 (2)	160

## References

[bb1] Akine, S., Taniguchi, T., Dong, W. K., Masubuchi, S. & Nabeshima, T. (2005). *J. Org. Chem.* **70**, 1704–1711. 10.1021/jo048030y 15730291

[bb2] Dong, W.-K., Ding, Y.-J., Luo, Y.-L., Yan, H.-B. & Wang, L. (2008). *Acta Cryst.* E**64**, o1636. 10.1107/S1600536808020692 PMC2962214 21203325

[bb3] Dong, W. K., Sun, Y. X., Zhang, Y. P., Li, L., He, X. N. & Tang, X. L. (2009*a*). *Inorg. Chim. Acta*, **362**, 117–124.

[bb4] Dong, W. K., Zhao, C. Y., Sun, Y. X., Tang, X. L. & He, X. N. (2009*b*). *Inorg. Chem. Commun.* **12**, 234–236.

[bb5] Sheldrick, G. M. (2008). *Acta Cryst.* A**64**, 112–122. 10.1107/S0108767307043930 18156677

[bb6] Siemens (1996). *SMART* and *SAINT* Siemens Analytical X-ray Instruments Inc., Madison, Wisconsin, USA.

[bb7] Yamada, S. (1999). *Coord. Chem. Rev.* **190**, 537–555.

